# Utilizing the theory of planned behavior to predict COVID-19 vaccination intention: A structural equational modeling approach

**DOI:** 10.1016/j.heliyon.2023.e17418

**Published:** 2023-06-17

**Authors:** Huy Nhuong Bui, Cong Doanh Duong, Van Quang Nguyen, Ngoc Xuan Vu, Son Tung Ha, Trung Thanh Le, Trong Nghia Vu

**Affiliations:** a National Economics University, Hanoi, Viet Nam; b College of Economics, Technology and Fisheries, Viet Nam; c TIMAS – Thang Long University, Hanoi, Viet Nam

**Keywords:** COVID-19 vaccination intention, Perceived benefits of COVID-19 vaccines, Theory of planned behavior

## Abstract

It is essential to achieve herd immunity in order to control the COVID-19 pandemic, and this requires a high level of vaccination rate. Despite the importance of vaccination, hesitancy and unwillingness in receiving the COVID-19 vaccine still exists. It is therefore crucial to comprehend the intentions of adults regarding COVID-19 vaccination, which is beneficial for establishing community immunity and an efficient future pandemic response. An online survey was administered to 2722 adults in Vietnam. Cronbach's alpha, exploratory factor analysis (EFA), and confirmatory factor analysis (CFA) were used to test the reliability and validity of the developed scales. Then, structural equational modeling (SEM) was employed to test correlations. This study found that favorable attitudes toward COVID-19 vaccines played the most important role in shaping adults' intention to receive these vaccines, followed by perceived behavioral control, perceived benefits of COVID-19 vaccines, and subjective norms. Concurrently, all three core dimensions of the theory of planned behavior mediated the link between the perceived benefits of COVID-19 vaccines and the intention to receive them. Also, there were significant differences between males and females in the way they formed this intention. The findings of this study offer valuable guidance for practitioners on how to encourage adults to receive COVID-19 vaccinations, as well as how to limit the transmission of the COVID-19 virus.

## 1 Introduction

The emergence of the novel coronavirus disease 2019 (COVID-19) was initially observed in Wuhan city, China, in late 2019, and it rapidly spread to become a global pandemic [[1], [2], [3]]. Confirmed cases and deaths have been reported in over 200 countries worldwide. According to the World Health Organization (WHO)'s report as of May 23, 2023 by that point there had been over 766 million cumulative cases and over 6.9 million deaths globally due to COVID-19 [4]. This pandemic has resulted in significant losses and widespread socioeconomic distress across the globe [5,6].

Since the early phase of the COVID-19 pandemic, a number of measures have been applied and practiced in attempts to restrain and contain the spread of the COVID-19 infection; these include social distancing, hand washing, face-mask wearing, border shutdowns, quarantine, and travel restrictions, among others [[7], [8], [9], [10], [11]]. As part of the ongoing measures to restrict the spread of the COVID-19 pandemic, vaccination played a crucial role in curbing and resolving the COVID-19 pandemic [[12], [13], [14]]. Thus, shortly after the outbreak of the disease was declared, there was an urgent call for COVID-19 vaccine research and development by the WHO [15]. The COVID-19 pandemic has spurred unprecedented efforts in vaccine research [[16], [17], [18]] and development in terms of speed and scale, resulting in the approval of several vaccines by the WHO, such as Moderna, Pfizer/BioNTech, and AstraZeneca [12]. Despite this progress, vaccine hesitancy remained a widespread issue [17,19], with vaccine refusal observed globally, hindering the high vaccination rates necessary to achieve herd immunity [20]. Failure to achieve this level of immunity due to vaccine refusal could impede COVID-19 containment [21]. Therefore, understanding people's intentions regarding COVID-19 vaccination was crucial for a successful vaccination campaign [3,22]. A body of research has been conducted in different countries, such as Hong Kong [22,23], the USA [11], Turkey [24], Canada [15], China [10,25] and many others [26,27]. These studies have explored the problems related to COVID-19 vaccine hesitancy and intentions. However, COVID-19 vaccination intentions and hesitancy have received scant attention in Southeast Asian countries, including Vietnam [12].

The theory of planned behavior (TPB) [28] has been considered to be one of the most influential theories that has been widely implemented to explain human behaviors [29], including behaviors related to COVID-19 vaccination [3,17,22]. Table 1 presented a concise summary of the findings from previous studies which applied the TPB to explain intentions/willingness to receive COVID-19 vaccines. In these studies, perceived benefits, an important dimension of the HBM, were integrated to be one of the most important predictors of intention to receive COVID-19 vaccines [22,[30], [31], [32], [33]], validating for the application of the TPB to explain how perceived benefits of COVID-19 vaccines affect COVID-19 vaccination intentions. In other words, our study aims to adopt the TPB to investigate the role of the perceived benefits of COVID-19 vaccines on attitudes towards COVID-19 vaccination (or attitudes), subjective norms, perceived behavioral control (perceived behavioral control), and COVID-19 vaccination intention among Vietnamese adults.

**Table 1:** Empirical contributions on applying the TPB to explain COVID-19 vaccination intention.

Authors (years)	Sampling and countries	Main influential factors	Remarks/key findings
Li, Lau [22]	11,141 parents, Hong Kong	Demographics, perceived susceptibility, perceived benefits, perceived barriers, cues to actions, attitudes, subjective norms, perceived behavioral control	higher levels of perceived susceptibility to COVID-19, perceived benefits, positive attitudes, subjective norms and lower levels of perceived barriers increased the level of COVID-19 vaccination intentions
Hayashi, Romanowich [34]	172 adult residents, United States	Demographic variables, attitudes, subjective norms, perceived behavioral control, locus of control, community benefit	Perceived behavioral control, attitude, and perceived community benefit are considered unique predictors
Wang, Li [35]	214 older adults, China	Perceived severity, perceived vulnerability, self-efficacy, response efficacy, response cost, attitudes, subject norms	Self-efficacy, subjective norms, perceived severity, and perceived vulnerability are determined as facilitator while response cost is identified a great obstacle
Ong, Prasetyo [32]	865 young adults, Philippines	Understanding of COVID-19 vaccine, self-efficacy, cues to action, perceived barriers, perceived benefit, perceived side-effects, attitude, perceived behavioral control, subjective norms	Understanding of the COVID-19 vaccines is the strongest predictor, followed by perceived barriers and perceived benefits
Shmueli [33]	398 adults, Israel	Social-demographic variables, health-related variables, HBM variables, and TPB variables	Intention to receive COVID-19 vaccines are sufficiently explained by integrating the variables of the HBM and the TPB
Ullah, Lin [36]	1034 young adults, Pakistan	Perceived infectability, attitude, subjective norms, perceived behavioral control, and fear of COVID-19	Attitude, subjective norms, perceived behavioral control, and fear of COVID-19 significantly mediate the relationship between perceived infectibility and COVID-19 vaccination intentions
Seddig, Maskileyson [37]	5044 adults, Germany	Fear of COVID-19, skepticism toward doctors and vaccines, COVID-19 conspiracy beliefs, trust variables and demographic variables	Attitude is the best predictor while normative and control beliefs did not predict COVID-19 vaccination intentions. Positive attitudes were bolstered by trust and fear of COVID-19 whilst negative attitudes were linked to acceptance of conspiracy theories and skepticism.

Vietnam has been identified as a suitable country for collecting data and applying the TPB to investigate how perceived benefits of COVID-19 vaccines can enhance the core components of the TPB and COVID-19 vaccination intention for the following reasons. Firstly, the effectiveness of the 5 K approach (Khẩu trang: mask wearing, Khử khuẩn: disinfection, Khoảng cách: social distancing, Không tụ tập đông người: No gatherings, and Khai báo y tế: health declaration) in controlling the spread of COVID-19 during the first three waves has been demonstrated in this country [12,38]. Secondly, during the time of the study, even though Vietnam was developing its own COVID-19 vaccine (Nanocovax), the majority of the Vietnamese population had only received one dose of a COVID-19 vaccine and they were waiting for international support in order to be vaccinated with their second and third doses of the vaccine [12]. Finally, although some prior studies have investigated the willingness to receive COVID-19 vaccines and the acceptance of vaccination in Southeast Asian countries, the results were mixed and inconsistent, and more importantly, these studies did not explore individuals' COVID-19 vaccination intention in the light of the TPB. For example, Duong and Antriyandarti [38] surveyed 2500 respondents in four countries in Southeast Asia (Vietnam, Indonesia, the Philippines, and Malaysia) to examine the role of vaccine brand on individuals' willingness to be vaccinated against COVID-19 with six COVID-19 vaccines. This research revealed that a higher percentage of respondents express willingness to be vaccinated with Pfizer, Moderna, and AstraZeneca vaccines compared to Sinopharm, Janssen, and Sputnik-V vaccines while the influence of influential factors on the willingness to receive vaccination varies in terms of both magnitude and direction, and this variability was dependent on the specific vaccine brands. Moreover, Duong, Nguyen [39] conducted a qualitative study in Vietnam and reported that people's attitudes toward COVID-19 vaccines were mixed and subject to change. These attitudes were not static and were influenced by individuals' risk-benefit self-assessment of vaccination.

### 1.1 The roles of the three core components in the TPB

The TPB was widely recognized as a significant theoretical framework that has been effectively utilized to research behavioral intentions [40]. According to this social psychology theory, behavioral intentions were a strong predictor of actual behavior [3,41], particularly in the context of reasoned and goal-directed actions [29]. Additionally, the TPB has demonstrated its effectiveness as a reliable predictor of both intentions and the subsequent behaviors related to receiving COVID-19 vaccines [3,22,[42], [43], [44]]. According to the TPB, intentions most closely predicted actual behaviors while behavioral intentions could be identified as a function of three variables: favorable or unfavorable perception and evaluation of a certain behavior (attitude toward behavior); perceptions of social pressure to carry out or not carry out the behavior (subjective norms); and the perception of the ease or difficulty of carrying out the behavior (perceived behavioral control) [40,45]. Some recent studies have also reported that COVID-19 vaccination intention was positively correlated with attitudes towards COVID-19 vaccination, subjective norms, and perceived behavioral control [3,22]. In the context of Vietnam, it was therefore hypothesized that COVID-19 vaccination intention could be positively driven by favorable attitudes towards COVID-19 vaccination (i.e. receiving a COVID-19 vaccine is attractive to me, receiving a COVID-19 vaccine would bring me great satisfaction), subjective norms (i.e., my friends think that I should have the COVID-19 vaccines, my parents think that I should have the COVID-19 vaccines), and perceived behavioral control (i.e., If I try, I can easily access and receive a COVID-19 vaccine).

H1 COVID-19 vaccination intention is positively interrelated with (a) attitudes towards COVID-19 vaccination; (b) subjective norms; and (c) perceived behavioral control.

Some studies that applied the TPB to explain the intention to conduct different behaviors have indicated that subjective norms can have a significant impact on attitudes toward behaviors, and on perceived behavioral control [46,47]. This was because the opinions and approval of surrounding people could increase our favorable perceptions of performing a certain behavior and how easy or difficult we perceive that it will be to carry out this behavior [40]. Moreover, studies have also found that perceived behavioral control can serve as a predictor of attitude toward behaviors [48,49]. Consequently, as well as testing the effect of the three components of the TPB (subjective norms, attitudes towards COVID-19 vaccination, and perceived behavioral control), this research also examined how subjective norms can increase attitudes towards COVID-19 vaccination and perceived behavioral control, as well as how perceived behavioral control could increase attitudes towards COVID-19 vaccination among Vietnamese adults.

H2 Subjective norms are positively interrelated with (a) attitudes towards COVID-19 vaccination and (b) perceived behavioral control.

H3 Perceived behavioral control is positively interrelated with attitudes towards COVID-19 vaccination.

### 1.2 The role of the perceived benefits of COVID-19 vaccines

Perceived benefits was a component of the HBM [50], which was a crucial theoretical framework that predicted whether an individual would engage in preventive health behavior such as receiving vaccinations, based on their beliefs. These beliefs included perceived susceptibility to the infection/disease, the perceived severity of becoming ill with this disease or infection, the perceived benefits of the behavior, the perceived barriers to carrying it out, and cues to action. According to the HBM, if a person perceived that adopting a specific health behavior would decrease their susceptibility or the severity of a health risk, they were more likely to adopt it, provided that the benefits outweighed the barriers and that they were prompted by internal or external cues [22,32]. Mir, Parveen [44] argued that the perceived BCV was that being vaccinated decreases the risk of becoming severely infected, which could result in a range of health risks and potentially life-threatening consequences. Among individuals who became infected with COVID-19 after receiving the vaccine, the need for hospitalization and medical care was generally lower than among those who have not been vaccinated [31]. Moreover, vaccinations provided social benefits by reducing the spread of COVID-19, thereby safeguarding society from a pandemic [42,51]. The majority of the respondents in Mir and Parveen's study agreed that having the vaccine was a good option for protecting themselves from COVID-19 infection [44]. Therefore, perceived benefits of COVID-19 vaccines could be determined as a central factor of COVID-19 vaccination intention [31,42,52], and the three core dimensions of the TPB also could act as mediators in the link between perceived benefits of COVID-19 vaccines and COVID-19 vaccination intention. The following hypotheses have been formulated to examine the effects of perceived benefits of COVID-19 vaccines on the attitudes towards COVID-19 vaccination, subjective norms, and perceived behavioral control of Vietnamese adults.

H4 Perceived benefits of COVID-19 vaccines are positive interrelated with (a) attitudes towards COVID-19 vaccination; (b) subjective norms; (c) perceived behavioral control; and (d) COVID-19 vaccination intention.

### 1.3 The role of gender

Males and females tended to behave in different ways as a result of their different paths in the socialization process, according to Gender Socialization Theory [53]. In a recent study, Dou, Yang [3] highlighted that there were notable variances between males and females in terms of their willingness to receive vaccines, with males being more inclined to have vaccines than females. This research tested the moderation effects of gender on the relationships between the TPB components and COVID-19 vaccination intention. In the context of Vietnam, it was hypothesized that the effects of the antecedents on COVID-19 vaccination intention couldbe different for males and females.

H5 There are differences in the impacts of (a) perceived benefits of COVID-19 vaccines, (b) attitudes towards COVID-19 vaccination, (c) subjective norms, and (d) perceived behavioral control on COVID-19 vaccination intention for males and females.

**Fig. 1:**
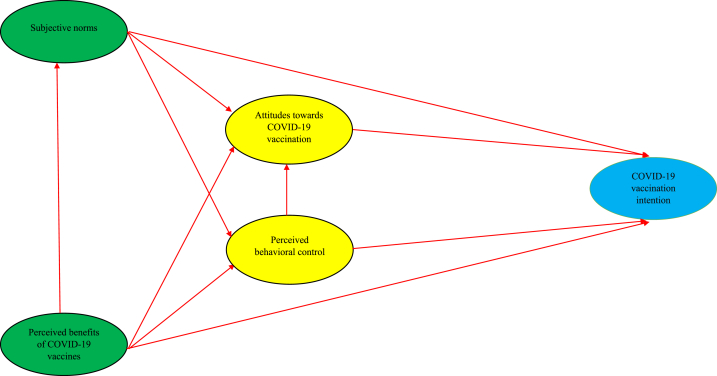
Theoretical model.

The conceptual framework is illustrated in Fig. 1.

## 2 Methods

### 2.1 Measures

In order to estimate the influences of perceived benefits of COVID-19 vaccines on COVID-19 vaccination intention via the three core components of the TPB, a questionnaire survey was employed in our study to gather the data. All of the measures (scales) used in our study were modified from prior studies. Particularly, the first three-item scale of COVID-19 vaccination intention was taken from the research of Chu and Liu [42], while the remaining items were taken from the research of Mir, Parveen [44], (i.e., “I try to get COVID-19 vaccines”, “My goal is to receive COVID-19 vaccines as soon as possible”). The five-item scale of attitudes towards COVID-19 vaccination (i.e., “Receiving a COVID-19 vaccine implies more advantages than disadvantages to me”) and the five-item scale of perceived behavioral control (i.e., “To receive a COVID-19 vaccine would be easy to me”) was modified from Mir, Parveen [44] and Liñán and Chen [54]. The first two items of the subjective norms scale were adapted from Chu and Liu [42], while the last three items of this scale were modified from Liñán and Chen [54] (i.e., “Most people who are important to me will get vaccinated for COVID-19”). Finally, the seven-item scale of perceived benefits of COVID-19 vaccines was adopted from Chu and Liu [42] and Coe, Elliott [26] (i.e., “COVID-19 vaccines will be effective in preventing COVID-19”, “COVID-19 vaccines are very safe”. All of the observations were scored from 1 to 7, representing “strongly disagree” to “strongly agree”, respectively.

The final section of the questionnaire survey asked for the demographic details of the participants, such as their gender, age, monthly income, and degree of education. As suggested by Lee, Chinna [55], and because the target audience for the survey was Vietnamese adults, all of the questionnaire items were initially translated from English to Vietnamese. Subsequently, to ensure the accuracy of the translation process, two language specialists re-translated the questionnaire items back to English and then compared the two versions.

### 2.2 Sample and procedure

Our study employed a convenience sampling method and an online-based survey in Vietnam to gather the data between 20 July and October 20, 2021. At that time, Vietnam was going through its fourth significant wave of the COVID-19 pandemic, and various restrictions and social distancing measures were in place [12]. As a result, an online-based survey using Google-Forms was deemed most suitable for collecting data during this period [6]. Our study was conducted in accordance with the Declaration of Helsinki, and it received ethical approval from the Research Ethics Committee of the Faculty of Economics at the College of Economics, Technology and Fisheries, Bac Ninh, Vietnam, and the Department of Research Management, National Economics University, Hanoi, Vietnam, with the reference number of CBQT1.2022.06. Additionally, all of the participants provided informed consent to participate in the survey, with the understanding that their participation was completely voluntary, that they had the option to withdraw from the study at any point they wished, and that the data were intended solely for academic purposes. Furthermore, each participant only had to spend around 15 min completing the questionnaire, and all of their information was kept completely confidential, and any identifying details removed.

Our online survey was distributed directly to personal email addresses and messaging platforms, including Facebook, Zalo, and Viber, to invite potential participants to complete the survey. Ultimately, 2722 individuals completed the questionnaire, resulting in a response rate of 30.2%. The demographic details of the respondents are as follows. Although the majority of the respondents were female (56%), most were aged between 18 and 28 years old, accounting for 60.7% of the total, followed by those aged 29–28 (21.0%), 39–48 (13%), 49–58 (3.8%), and over 59 years old (1.6%). Over half (57.7%) of the respondents reported earning less than 10 million VND per month. In terms of education and marital status, 68.6% of the participants held a bachelor's degree, and 66.3% were single. With regard to vaccine preference, 29.1% of the respondents indicated a preference for the AstraZeneca vaccine, followed by Pfizer (19.5%), Moderna (15.3%), and Sinopharm (6.8%). Finally, 890 respondents reported receiving at least one dose of a COVID-19 vaccine, accounting for 32.7%. In fact, during the time of the study, even though Vietnam was developing its own COVID-19 vaccine (Nanocovax), the majority of the Vietnamese population have still not been vaccinated or only received one-dose of COVID-19 vaccines and were waiting for international support sources in order to be vaccinated with the second and third dose of COVID-19 vaccines [12]. Thus, the concept of “COVID-19 vaccination intention” not only reflects intention to receive the first dose of COVID-19 vaccine, but also shows individuals' intentions to getting vaccinated with the second and third dose of COVID-19 vaccine.

### 2.3 Analytical approach

In the first step, the reliability and validity of the scales were tested using Cronbach's alpha, exploratory factor analysis (EFA), and confirmatory factor analysis (CFA). The criteria of indices of model fit, standardized regression weight, AVE (Average Variance Extracted) and CR (Composite Reliability) were employed in these analyses, as suggested by some prior studies [[56], [57], [58]]. Additionally, common method bias (CMB) was then used to affirm the absence of a threat to the interpretation of the findings [59]. Next, to examine both direct and indirect associations in the theoretical model, we employed structural equation modeling (SEM) using AMOS 24.0. In this step, we followed the recommendations of Hayes and Matthes [60] to use Gaskin's (2019) plugin with 5000 bootstrapping samples to test the indirect impacts.

## 3 Results

### 3.1 Testing normality and scale assessment

All scales showed good internal consistency reliability, with Cronbach's alpha values higher than 0.63 (the lowest value was 0.896) and Corrected Item-Total correlations greater than 0.3 for each observed variable [61] (see Table 2). We conducted an EFA with all the items but found that one item (COVID-19 vaccination intention2 “I actually get vaccinated for COVID-19”) had a factor loading below 0.5 (λ_COVID-19 vaccination intention_ = 0.420). We removed this item and conducted a second EFA, which revealed five factors with a total extracted variance of 73.997%. The KMO value was 0.969, and all items had factor loadings higher than 0.5 [62].

**Table 2:** Reliability and discriminant validity of constructs.

	Mean	SD	α	CR	AVE	Bivariate correlations				
						(1)	(2)	(3)	(4)	(5)
1. Perceived benefits of COVID-19 vaccines	5.183	1.241	0.936	0.934	0.671	**0.819**				
2. COVID-19 vaccination intention	4.901	1.257	0.896	0.892	0.546	0.626**	**0.739**			
3. Attitudes towards COVID-19 vaccination	5.195	1.323	0.933	0.930	0.726	0.714**	0.698**	**0.852**		
4. Subjective norms	5.105	1.416	0.946	0.944	0.772	0.596**	0.627**	0.663**	**0.879**	
5. Perceived behavioral control	4.657	1.374	0.914	0.914	0.681	0.527**	0.625**	0.587**	0.556**	**0.825**

**Notes**: N = 2722; **α**: Cronbach's alpha, **p < 0.01.

**Fig. 2:**
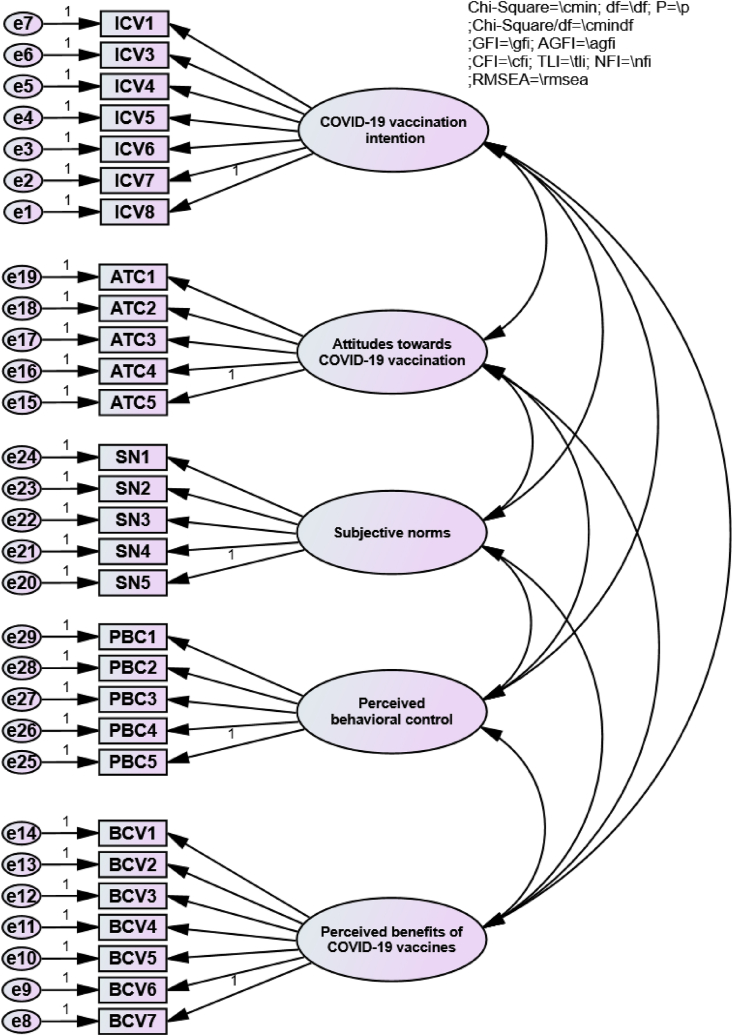
Standardized measurement model for the relationships between observed indicators and the latent variables.

Based on the results of the CFA, the fit indices suggested a good fit between the model and the data (see Fig. 2). The chi-square test was significant (χ^2^ (355) = 2968.836), but this was often the case with large sample sizes, indicating that other fit indices should be examined. The chi-square/df ratio was 8.363, which is below the recommended threshold of 10, indicating an acceptable fit [63]. The GFI and AGFI indices were both above their respective thresholds of 0.9 and 0.8, indicating a good fit. The CFI, TLI, and NFI indices were all above 0.9, indicating a good fit. Finally, the RMSEA index was 0.052, which was below the recommended threshold of 0.08, indicating a good fit. Table 2 also presented the construct reliability and discriminant validity. The AVE and CR values were also evaluated to test the reliability and validity of the scales. The AVE values were all above 0.5, indicating that each scale accounts for more than 50% of the variance in its respective items [64]. The CR values were all greater than 0.6, indicating high internal consistency reliability [65]. Additionally, the square roots of AVEs were all higher than the inter-construct correlations, indicating good discriminant validity. As a results, all the scales demonstrated their reliability and validity [66].

Additionally, as all the participants completed the questionnaires themselves, we used both EFA and CFA analyses to assess whether CMB could affect the interpretation of our results. Initially, we conducted Harman's single-factor test to identify any CMB. The outcome revealed that only 28.63% of the variance was accounted for by the first factor [67]. Moreover, the observed variables of the four constructs were constrained using a single-factor measurement model. However, the results of the single-factor CFA demonstrated that the fitness indices did not meet the desired level of acceptability: χ^2^ (434) = 23542.781; GFI = 0.524; AGFI = 0.456; CFI = 0.667; TLI = 0.643; NFI = 0.663; RMSEA = 0.140. Consequently, it appeared that CMB did not present a significant issue for the interpretation of our findings [68,69].

### 3.2 Structural equation modeling

**Fig. 3:**
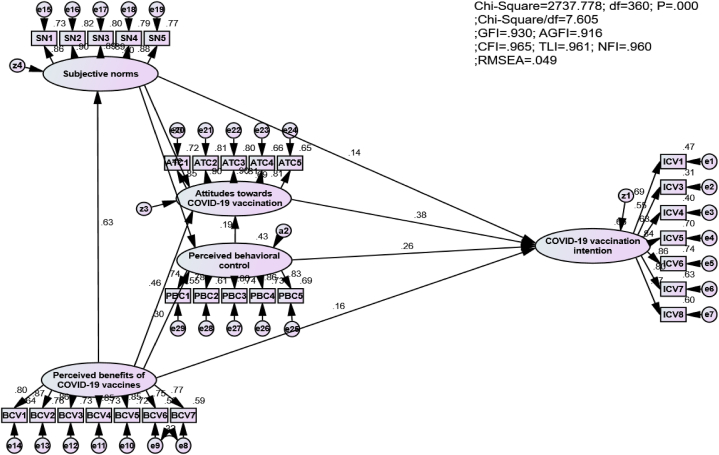
Structural equation modeling.

The results of the SEM analysis indicated that the model achieved a high level of fit (see Fig. 3). To specify, χ^2^ (360) = 2737.778; Chi-square/df = 7.605; p < 0.01; GFI = 0.930 > 0.9; AGFI = 0.916 > 0.9; CFI = 0.965 > 0.9; TLI = 0.961 > 0.9; and RMSEA = 0.049 < 0.8 [65]. The Square Multiple Correlations (R^2^) of COVID-19 vaccination intention, attitudes towards COVID-19 vaccination, subjective norms, and perceived behavioral control were 0.665, 0.687, 0.396, and 0.426, respectively. The results of testing the hypotheses were summarized in Table 3. First, the results confirmed that the three core antecedents in the TPB played an important role in shaping COVID-19 vaccination intention. Indeed, COVID-19 vaccination intention was significantly affected by attitudes towards COVID-19 vaccination (H1a: β = 0.344; p-value <0.001), subjective norms (H1b: β = 0.121; p-value <0.001) and perceived behavioral control (H1c: β = 0.215; p-value <0.001). In addition, subjective norms were significantly associated with attitudes towards COVID-19 vaccination (H2a: β = 0.285; p-value <0.001) and perceived behavioral control (H2b: β = 0.427; p-value <0.001) while perceived behavioral control significantly influenced attitudes towards COVID-19 vaccination (H3: β = 0.179; p-value <0.001).

**Table 3:** Results of hypothesis testing.

Hypotheses				Estimate	S.E.	C.R.	*P*-value	Results
H1a	attitudes towards COVID-19 vaccination	→	COVID-19 vaccination intention	0.344	0.026	13.418	***	Supported
H1b	subjective norms	→	COVID-19 vaccination intention	0.121	0.018	6.599	***	Supported
H1c	perceived behavioral control	→	COVID-19 vaccination intention	0.215	0.017	12.359	***	Supported
H2a	subjective norms	→	attitudes towards COVID-19 vaccination	0.285	0.018	15.826	***	Supported
H2b	subjective norms	→	perceived behavioral control	0.427	0.023	18.393	***	Supported
H3	perceived behavioral control	→	attitudes towards COVID-19 vaccination	0.179	0.017	10.636	***	Supported
H4a	perceived benefits of COVID-19 vaccines	→	attitudes towards COVID-19 vaccination	0.498	0.022	23.123	***	Supported
H4b	perceived benefits of COVID-19 vaccines	→	subjective norms	0.723	0.024	30.739	***	Supported
H4c	perceived benefits of COVID-19 vaccines	→	perceived behavioral control	0.346	0.026	13.085	***	Supported
H4d	perceived benefits of COVID-19 vaccines	→	COVID-19 vaccination intention	0.154	0.023	6.733	***	Supported

Notes: N = 2722, ***p < 0.001.

In terms of the impact of perceived benefits of COVID-19 vaccine, results revealed that these significantly affected attitudes towards COVID-19 vaccination (H4a: β = 0.498; p-value <0.001), subjective norms (H4b: β = 0.723; p-value <0.001), perceived behavioral control (H4c: β = 0.346; p-value <0.001), and COVID-19 vaccination intention (H4d: β = 0.154; p-value <0.001). Besides testing the direct effects, our study adopted Gaskin's (2019) plugin, based on the suggestions of Hayes and Matthes [60], with 5000 bootstrapping samples to test the indirect impacts. The results reported that perceived benefits of COVID-19 vaccines not only had indirect effects on COVID-19 vaccination intention through the three elements of the TPB, including attitudes towards COVID-19 vaccination (β = 0.171; p-value <0.001), subjective norms (β = 0.088; p-value <0.001), and perceived behavioral control (β = 0.074; p-value <0.001), but it also serially and indirectly affected COVID-19 vaccination intention via the serial perceived benefits of COVID-19 vaccines-subjective norms-perceived behavioral control-attitudes towards COVID-19 vaccination-COVID-19 vaccination intention path (β = 0.019; p-value <0.001) (see Table 4).

**Table 4:** Results of estimating indirect effects.

Indirect paths									Estimates	*P*-value
subjective norms	→	perceived behavioral control	→	attitudes towards COVID-19 vaccination					0.076	***
subjective norms	→	attitudes towards COVID-19 vaccination	→	COVID-19 vaccination intention					0.098	***
subjective norms	→	perceived behavioral control	→	COVID-19 vaccination intention					0.092	***
perceived behavioral control	→	attitudes towards COVID-19 vaccination	→	COVID-19 vaccination intention					0.061	***
perceived benefits of COVID-19 vaccines	→	subjective norms	→	attitudes towards COVID-19 vaccination					0.026	***
perceived benefits of COVID-19 vaccines	→	subjective norms	→	perceived behavioral control					0.039	***
perceived benefits of COVID-19 vaccines	→	perceived behavioral control	→	attitudes towards COVID-19 vaccination					0.062	***
perceived benefits of COVID-19 vaccines	→	attitudes towards COVID-19 vaccination	→	COVID-19 vaccination intention					0.171	***
perceived benefits of COVID-19 vaccines	→	subjective norms	→	COVID-19 vaccination intention					0.088	***
perceived benefits of COVID-19 vaccines	→	perceived behavioral control	→	COVID-19 vaccination intention					0.074	***
perceived benefits of COVID-19 vaccines	→	subjective norms	→	perceived behavioral control	→	attitudes towards COVID-19 vaccination			0.055	***
perceived benefits of COVID-19 vaccines	→	subjective norms	→	attitudes towards COVID-19 vaccination	→	COVID-19 vaccination intention			0.071	***
perceived benefits of COVID-19 vaccines	→	subjective norms	→	perceived behavioral control	→	COVID-19 vaccination intention			0.066	***
perceived benefits of COVID-19 vaccines	→	subjective norms	→	perceived behavioral control	→	attitudes towards COVID-19 vaccination	→	COVID-19 vaccination intention	0.019	***

Notes: N = 2722. ***p < 0.001.

Table 5 illustrated the differences in the effects of factors on males and females' COVID-19 vaccination intention. Indeed, while the effects of attitudes towards COVID-19 vaccination (H5a: β = 0.320; p-value <0.001; β = 0.270 p-value <0.001, respectively) and perceived behavioral control (H5c: β = 0.258; p-value <0.001; β = 0.205 p-value <0.001, respectively) on the COVID-19 vaccination intention of females were much higher than on the COVID-19 vaccination intention of males. The impacts of subjective norms (H5b: β = 0.147; p-value <0.001; β = 0183, p-value <0.001, respectively) and the perceived benefits of COVID-19 vaccines (H5d: β = 0.132; p-value <0.001; β = 0.196, p-value <0.001, respectively) on females' COVID-19 vaccination intention were much lower than on males’ COVID-19 vaccination intention.

**Table 5:** The differences between males and females in terms of the effects of factors and COVID-19 vaccination intention.

Hypotheses	Variables	COVID-19 vaccination intention				Results
		Males		Females		
	Intercept	0.599***	(0.170)	0.592***	(0.097)	
H5a	attitudes towards COVID-19 vaccination	0.270***	(0.028)	0.320***	(0.026)	Supported
H5b	subjective norms	0.183***	(0.023)	0.147***	(0.021)	Supported
H5c	perceived behavioral control	0.205***	(0.021)	0.258***	(0.020)	Supported
H5d	perceived benefits of COVID-19 vaccines	0.196***	(0.028)	0.132***	(0.025)	Supported
N (observations)		1197		1525		
ΔF		442.196***		547.567***		
R^2^		0.597		0.590		

*Note*: *P*-values are provided in brackets, ***p < 0.001.

## 4 Discussion

This study applied the TPB and SEM analyses to predict COVID-19 vaccination intention, and to test the effect of perceived benefits of COVID-19 vaccines on COVID-19 vaccination intention via the three core dimensions of TPB. Using the sample of 2722 adults, our study firstly found that attitudes towards COVID-19 vaccination was the most influential factor with regard to the formation of COVID-19 vaccination intention. This meant that a favorable attitudes towards COVID-19 vaccination is necessary to foster citizens' COVID-19 vaccination intention [10,38]. In other words, to promote citizens' engagement with COVID-19 vaccines, measures to increase positive attitudes towards COVID-19 vaccination should be introduced. Secondly, perceived behavioral control was found to have a strong and positive effect on COVID-19 vaccination intention. This demonstrated that adults' perceived ability and ease of receiving a COVID-19 vaccine significantly contribute to their COVID-19 vaccination intention. At the time of this study, even though the COVID-19 vaccines were not available for over 100 million Vietnamese citizens, many individuals could easily access COVID-19 vaccines as Vietnam received support from the international community thanks to the success of the COVID-19 vaccine diplomacy policy [12,70]. From our participants, 890 of the 2722 adults had already received at least one dose of the vaccine. In addition, subjective norms significantly and positively affected COVID-19 vaccination intention. This aligned with the fact that in a collectivism culture like Vietnam, the decision to conduct a behavior was often influenced by other people. Indeed, opinions/approval from surrounding people (parents, brothers, sisters, friends, and others) were strongly related to individuals' COVID-19 vaccination intention. These findings were in line with the study of Chu, Gupta [43], although this study adopted the TPB to identify individuals' intentions to receive the influenza vaccine. It revealed that all three core dimensions of the TPB were positively associated with these intentions. The findings of our study were different from those of prior studies conducted in Southeast Asia, as well as in Vietnam. For example, while our study applied the TPB to explain how perceived benefits of COVID-19 vaccines could help increase individuals' attitudes towards COVID-19 vaccination, subjective norms, perceived behavioral control, and COVID-19 vaccination intention, prior studies either examined the other drivers of COVID-19 vaccine acceptance with various degrees of pandemic severity [38], or aimed to understand the beliefs and attitudes of South-Asian individuals toward COVID-19 vaccinations [39,71,72]. Particularly, the current study reported the direct and indirect effects of perceived benefits of COVID-19 vaccines on COVID-19 vaccination intention among adults in Vietnam through three core components of the TPB, such as attitudes towards COVID-19 vaccination, subjective norms, perceived behavioral control. However, Chew, Cheong [72] based on the sample of healthcare workers, revealed that a majority of healthcare workers in Asia express willingness to receive COVID-19 vaccination, citing perceived susceptibility to COVID-19, low potential risk of vaccine harm, and pro-socialness as the main driving factors while Hawlader, Rahman [71] revealed that The willingness to receive COVID-19 vaccination among respondents was found to be 65%, 66%, 72%, and 74% for Bangladesh, India, Pakistan, and Nepal, respectively. Significant factors that influenced respondents’ intentions included the perceived destructive impact of COVID-19, positive perception of vaccines, and concerns about possible side effects.

Noticeably, perceived benefits of COVID-19 vaccines have been determined as a key precursor of attitudes towards COVID-19 vaccination, subjective norms, perceived behavioral control, and COVID-19 vaccination intention. Therefore, perceptions related to the effectiveness of COVID-19 vaccines in preventing COVID-19 infections, the benefits of vaccination for the health of others in the community, the safety of COVID-19 vaccines, and the prospect of things going back to normal are crucial factors in individuals’ favorable attitudes towards COVID-19 vaccination and higher COVID-19 vaccination intention [26,42]. Importantly, these results illustrated those attitudes towards COVID-19 vaccination, subjective norms, and perceived behavioral control acted as partial mediators in the link between perceived benefits of COVID-19 vaccines and COVID-19 vaccination intention. In other words, the three core antecedents of the TPB (attitudes towards COVID-19 vaccination, subjective norms, and perceived behavioral control) could transfer the effects of perceived benefits of COVID-19 vaccines on them into COVID-19 vaccination intention. Finally, our results found significant differences in the impacts of attitudes towards COVID-19 vaccination, subjective norms, perceived behavioral control and perceived benefits of COVID-19 vaccines on COVID-19 vaccination intention. For example, males tended to be more influenced by the opinions of other people than females in terms of receiving COVID-19 vaccines.

### 4.1 Strengths of the current study

Our research offered important strengths. One significant contribution was that it adds to the existing literature on COVID-19 vaccine hesitancy by being one of the first studies conducted in Southeast Asia. Our study shed new light on the intentions to receive the COVID-19 vaccine among adults in this region, thereby expanding our understanding of this important public health issue. Moreover, although prior studies have adopted various theories to explain individuals' COVID-19 vaccination intention, such as the integrative model of behavioral prediction [73] and health behavior theories [42], this study was one of the earliest to employ the TPB [28] to explain adults’ COVID-19 vaccination intention in the context of Vietnam. Our study contributed to our knowledge by illustrating that all three attitudinal antecedents in the TPB make significant contributions to explaining why individuals have COVID-19 vaccination intention. Furthermore, this study was the first to test the mediating roles of the three core dimensions in the TPB (attitudes towards COVID-19 vaccination, subjective norms, and perceived behavioral control) in the link between perceived benefits of COVID-19 vaccines and COVID-19 vaccination intention.

### 4.2 Policy implications

The results of this research provided practical guidance for policymakers, practitioners, and governments to promote COVID-19 vaccination intentions and behaviors among citizens and to help to control the spread of the COVID-19 pandemic through vaccination. In fact, to foster adults' COVID-19 vaccination intention and vaccination behaviors, practitioners should introduce appropriate measures to promote favorable attitudes towards COVID-19 vaccination as well as to help adults access COVID-19 vaccines more easily to increase their perceived behavioral control. Concurrently, our study showed that the opinions/approval of surrounding people are very important for individuals' decision to have COVID-19 vaccinations. Therefore, suitable communication or marketing measures to increase citizens’ awareness of the benefits of COVID-19 vaccines, for both their own and community health, as well as the safety of COVID-19 vaccines, contribute to forming positive intentions and engagement with COVID-19 vaccines. Lastly, the gender issue should be appropriately addressed as antecedents of COVID-19 vaccination intention impact males and females differently.

## 5 Limitations

It is important to acknowledge the limitations of our study. Firstly, our sampling method relied on an online survey. Although our sample size was considerable, more than 60% of the sample were aged between 18 and 28 years old as a result of the online nature of the research, while the individuals most vulnerable to the serious side effects of COVID-19 are over the age of 65 [74,75]. Thus, this was also considered to be a limitation of our study. Future studies should employ random sampling methods to improve the representativeness of the research sample as well as to focus on elderly people, who are seriously affected by COVID-19. Secondly, Vietnam had limited access to COVID-19 vaccines during the period of our study, which restricted our ability to explore adults’ intentions to receive the COVID-19 vaccines. Future research should examine the intention-behavior link in individuals' decision to be vaccinated and investigate the factors underlying this decision. Finally, the cross-sectional design was another limitation of our study. Carlson and Morrison [76] argued that the main drawback of a cross-sectional study design was that since the exposure and outcome were evaluated at the same time, there is likely to be no indication of a causal relationship between them. The number of adults who received at least one dose of the COVID-19 vaccine showed the limitation of this research as it reflected their actual behaviors regarding COVID-19 vaccination instead of their behavioral intention. A longitudinal design should be employed in future research to examine the effects of different factors on intentions and behaviors related to receiving COVID-19 vaccines.

## 6 Conclusion

The TPB has been applied in the current study to estimate the direct and indirect effects of perceived benefits of COVID-19 vaccines on the three core elements of the TPB, through individuals' attitudes towards COVID-19 vaccination, subjective norms, perceived behavioral control, and COVID-19 vaccination intention as well as the gender differences in these relationships. Based on our study's findings, it is clear that attitudes towards COVID-19 vaccination played a critical role in shaping the COVID-19 vaccination intention in adults, followed by perceived behavioral control, perceived benefits of COVID-19 vaccines, and subjective norms. These findings offered valuable insights for practitioners seeking to encourage vaccination uptake and limit the transmission of the COVID-19 pandemic. Furthermore, our study highlighted the significant differences between males and females in how they form the intention to have COVID-19 vaccines. This underscored the importance of tailoring public health campaigns to specific demographic groups to maximize their impact. Overall, our study added to the growing body of research on the TPB and its application to health behavior, specifically related to COVID-19 vaccination uptake among Vietnamese adults. Our findings provided practical recommendations for practitioners seeking to increase vaccination rates and help curb the spread of the COVID-19 pandemic.

## Author contribution statement

Huy Nhuong Bui: Conceived and designed the experiments; Performed the experiments; Analyzed and interpreted the data.

Cong Doanh Duong: Performed the experiments; Analyzed and interpreted the data; Wrote the paper.

Van Quang Nguyen; Trung Thanh Le; Trong Nghia Vu; Ngoc Xuan Vu: Contributed reagents, materials, analysis tools or data.

Son Tung Ha: Analyzed and interpreted the data; Contributed reagents, materials, analysis tools or data.

## Data availability statement

Data will be made available on request.

## Declaration of interest's statement

The authors declare no conflict of interest.

## Declaration of competing interest

The authors declare that they have no known competing financial interests or personal relationships that could have appeared to influence the work reported in this paper.
